# Racial differences in coping strategies for people living with ALS and their caregivers

**DOI:** 10.1093/geroni/igaf122.2590

**Published:** 2025-12-31

**Authors:** Omar Guerrero, Maame-Owusua Boateng, Ashley Nurse, Christina Pantzer, Asia Beason, Avery DeWitt, Joan Monin, Chelsey Carter

**Affiliations:** Yale University, New Haven, Connecticut, United States; Yale School of Public Health, New Haven, Connecticut, United States; Yale School of Public Health, New Haven, Connecticut, United States; Yale University, New Haven, Connecticut, United States; Yale School of Public Health, New Haven, Connecticut, United States; Yale School of Public Health, New Haven, Connecticut, United States; Yale, Yale, Connecticut, United States; Yale School of Public Health, New Haven, Connecticut, United States

## Abstract

People living with amyotrophic lateral sclerosis (ALS) and their caregivers face negative psychological distress. No research to our knowledge has examined the unique coping experiences of Black/Other ALS dyads. We conducted an on-line, self-report, cross-sectional study of the experiences of people living with ALS (N = 74; 60 White; 14 Black/Other) and caregivers of people living with ALS (N = 77; 57 White; 20 Black/Other). Both samples completed the Brief Cope (Carver, 1997), and we explored differences between the White and Black/Other participants for each sub-scale. We found that Black/Other caregivers (M = 2.75, SD = 1.05) used humor as a coping style more than White caregivers (M = 2.24, SD=.83; F(76)=4.93, p=.029). We also found that Black/Other people living with ALS (M = 2.18, SD=.87) reported using more religious coping than White people living with ALS (M = 1.64, SD=.55; F(73)=8.46, p=.005). Also, Black/Other people living with ALS (M = 1.68, SD=.75) used more positive coping than White people living with ALS (M = 1.24, SD=.45; F(73)=8.19). Results of this exploratory analysis suggest that future studies with larger samples replicate these findings. If replicated, these findings would have implications for the design of supportive psychoeducational interventions tailored to the needs of Black/Other caregiving dyads living with ALS. We highlight the need for advancing health equity approaches for other rare, and neurodegenerative diseases. .

